# Strong Convergence of a Monotone Projection Algorithm in a Banach Space

**DOI:** 10.1155/2013/764909

**Published:** 2013-11-21

**Authors:** Songtao Lv

**Affiliations:** School of Mathematics and Information Science, Shangqiu Normal University, Shangqiu 476000, China

## Abstract

In this paper, a common solution problem is investigated based on a Bregman projection. Strong convergence of the monotone projection algorithm for monotone operators and bifunctions is obtained in a reflexive Banach space.

## 1. Introduction 

Fixed point theory as an important branch of nonlinear analysis theory has been applied in the study of nonlinear phenomena. Lots of problems arising in economics, engineering, and physics can be studied based on fixed point techniques; see [1–21] and the references therein. Many well-known problems can be studied by using algorithms which are iterative in their nature. As an example, in computer tomography with limited data, each piece of information implies the existence of a convex set *C*_*m*_ in which the required solution lies. The problem of finding a point in the intersection ∩_*m*=1_^*N*^ *C*_*m*_, where *N* ≥ 1 is some positive integer, is then of crucial interest, and it cannot be usually solved directly. Therefore, an iterative algorithm must be used to approximate such point. The well-known convex feasibility problem which captures applications in various disciplines such as image restoration and radiation therapy treatment planning is to find a point in the intersection of common fixed point sets of a family of nonlinear mappings see, for example, [22–28].

Interest in monotone operators stems mainly from their firm connection with equations of evolution which is an important class of nonlinear operators. It is known that many physically significant problems can be modelled by initial value problems of the form:

(1) $$\begin{array}{c} x^{\prime }\left(t\right)+Ax\left(t\right)=0,\;x\left(0\right)=x_{0}, \end{array}$$

where *A* is an monotone operator in an appropriate Banach space. Typical examples, where such evolution equations occur, can be found in the heat, wave, or Schrödinger's equations. If *x*(*t*) is dependent on *t*, then (1) is reduced to

(2) $$\begin{array}{c} Au=0, \end{array}$$

whose solutions correspond to the equilibrium points of the system (1). Consequently, considerable research efforts have been devoted, especially within the past 40 years or so, to methods for finding approximate solutions (when they exist) of (2). An early fundamental result in the theory of monotone operators, due to Browder [29], states that the initial value problem (1) is solvable if *A* is locally Lipschitz and accretive on *E*.

The Krasnoselskii-Mann iterative algorithm is efficient for treating zero points of monotone operators. However, it is weak convergence only. In many disciplines, including economics, image recovery, and control theory, problems arise in infinite dimension spaces. In such problems, strong convergence (norm convergence) is often much more desirable than weak convergence for it translates the physically tangible property that the energy ||*x*_*n*_ − *x*|| of the error between the iterate *x*_*n*_ and the solution *x* eventually becomes arbitrarily small. The importance of strong convergence is also underlined, where a convex function *f* is minimized via the proximal-point algorithm; it is shown that the rate of convergence of the value sequence {*f*(*x*_*n*_)} is better when {*x*_*n*_} converges strongly than when it converges weakly. Such properties have a direct impact when the process is executed directly in the underlying infinite dimensional space. Projection methods which were first introduced by Haugazeau [30] have been considered for treating zero points of monotone operators. The advantage of projection methods is that strong convergence of iterative sequences can be guaranteed without any compact assumptions.

In this paper, we study common zero points of a family of maximal monotone operators and common solutions of a system of equilibrium problems based on the Bregman projection. Strong convergence of the monotone projection algorithm is obtained in a reflexive Banach space. 

## 2. Preliminaries

Let *E* be a real Banach space, E* the dual space of *E*, and *C* a nonempty subset of *E*. Let *f* be a bifunction from *C* × *C* to ℝ, where ℝ denotes the set of real numbers. Recall the following equilibrium problem. Find $\bar{x}\in C$ such that

(3) $$\begin{array}{c} f\left(\bar{x}y\right)\geq 0,\;\forall y\in C. \end{array}$$

We use *EP*⁡(*f*) to denote the solution set of the equilibrium problem (3). That is,

(4) $$\begin{array}{c} \mathrm{EP}\left(f\right)=\left\{p\in C:f\left(p,y\right)\geq 0,\forall y\in C\right\}. \end{array}$$

Given a mapping *B* : *C* → E*, let

(5) $$\begin{array}{c} f\left(x,y\right)=\langle Bx,y-x\rangle ,\;\forall x,y\in C. \end{array}$$

Then, $\bar{x}\in \mathrm{EP}(f)$ if and only if $\bar{x}$ is a solution of the following variational inequality. Find $\bar{x}$ such that

(6) $$\begin{array}{c} \langle B\bar{x}y-\bar{x}\rangle \geq 0,\;\;\forall y\in C. \end{array}$$

In order to study the solution of the equilibrium problem (3), we assume that *f* satisfies the following conditions:

(A1) *f*(*x*, *x*) = 0, for  all  *x* ∈ *C*;
(A2) *f* is monotone; that is, *f*(*x*, *y*) + *f*(*y*, *x*) ≤ 0, for  all  *x*, *y* ∈ *C*;
(A3) (7) $$\begin{array}{c} \underset{t↓0}{\mathrm{limsup}}\;f\left(tz+\left(1-t\right)x,y\right)\leq f\left(x,y\right),\;\forall x,y,z\in C; \end{array}$$
(A4) for each *x* ∈ *C*, *y* ↦ *f*(*x*, *y*) is convex and weakly lower semicontinuous.

For any convex function *g* : *E* → (−*∞*, +*∞*], we denote the domain of *g* by Dom⁡(*g*) = {*x* ∈ *E* : *g*(*x*) < *∞*}. For any *x* ∈ int⁡ Dom⁡(*g*) and *y* ∈ *E*, we denote by *g* ^0^(*x*, *y*) the right-hand derivative of *g* at *x* in the direction *y*; that is,

(8) $$\begin{array}{c} g^{0}\left(x,y\right)=\underset{t\to 0^{+}}{\lim}\;\frac{g\left(x+ty\right)-g\left(x\right)}{t}. \end{array}$$

The function *g* is said to be Gâteaux differentiable at *x* if lim⁡_*t*→0^+^_ ((*g*(*x* + *ty*) − *g*(*x*))/*t*) exists for any *y*. In this case, *g* ^0^(*x*, *y*) coincides with ∇*g*(*x*), the value of the gradient ∇*g* of *g* at *x*. The function *g* is said to be Gâteaux differentiable if it is Gâteaux differentiable everywhere. The function *g* is said to be Fréchet differentiable at *x* if the limit is attained uniformly in ||*y*|| = 1. The function *g* is said to be Fréchet differentiable if it is Fréchet differentiable everywhere. It is well known that if a continuous convex function *g* : *E* → ℝ is Gâteaux differentiable, then ∇*g* is norm-to-weak* continuous. If *g* : *E* → ℝ is Fréchet differentiable, then ∇*g* is norm-to-norm continuous; for more details, see [31] and the references therein; for more details, see [32] and the references therein. The function *g* is said to be strongly coercive [32] if lim⁡_||*x*||→*∞*_ (*f*(*x*)/||*x*||) = *∞*. It is said to be bounded on bounded subsets of *E* if *g*(*B*) is bounded for each bounded subset *B* of *E*. Let *E* be a reflexive Banach space. For any proper, lower semicontinuous, and convex function: *g* : *E* → (−*∞*, +*∞*], the conjugate function g* of *g* is defined by

(9) $$\begin{array}{c} g^{*}\left(x^{*}\right)=\underset{x\in E}{\sup}\left\{\langle x,x^{*}\rangle -g\left(x\right)\right\},\;\forall x^{*}\in E^{*}. \end{array}$$

It is well known that *g*(*x*) + g*(x*) ≥ 〈*x*, x*〉 for all (*x*, x*) ∈ *E* × E*. It is also known that (*x*, x*)∈∂*g*, where ∂*g* is the subdifferential of *g*, is equivalent to

(10) $$\begin{array}{c} g\left(x\right)+g^{*}\left(x^{*}\right)=\langle x,x^{*}\rangle . \end{array}$$

If *g* is a proper, lower semicontinuous, and convex function, then g* is a proper, weak* lower semicontinuous, and convex function.

Next, we recall some facts about Bregman distance. Let *g* : *E* → ℝ be a convex and Gâteaux differentiable function. Then, Bregman distance corresponding to *g* is the function *D*_*g*_ : *E* × *E* → ℝ defined by

(11) $$\begin{array}{c} D_{g}\left(x,y\right)=g\left(x\right)-g\left(y\right)-\langle x-y,\nabla g\left(y\right)\rangle ,\;\forall x,y\in E. \end{array}$$

It is clear that *D*_*g*_(*x*, *y*) ≥ 0 for all *x*, *y* ∈ *E*. If *E* is smooth and *g*(*x*) = ||*x*||^2^ for all *x* ∈ *E*, we obtain that ∇*g*(*x*) = 2*Jx*, where *J* is the generalized duality mapping. If *C* is a nonempty, closed and convex subset of a reflexive Banach space *E* and *g* is a strongly coercive Bregman function, then for each *x* ∈ *E*, there exists a unique *x*_0_ ∈ *C* such that *D*_*g*_(*x*_0_, *x*) = min⁡_*y*∈*C*_ *D*_*g*_(*y*, *x*). Bregman projection Proj_*C*_^*g*^ from *E* onto *C* is defined by Proj_*C*_^*g*^ *x* = *x*_0_ for all *x* ∈ *E*. It is also well known [32] that Proj_*C*_^*g*^ has the following property:

(12) Dg(y,ProjCgx)+Dg(ProjCgx,x)≤Dg(y,x),∀y∈C,  x∈E.

Let *B*_*r*_ : = {*z* ∈ *E* : ||*z*|| < *r*}. A function *g* : *E* → ℝ is said to be uniformly convex on bounded subsets of *E* if *f*_*r*_(*t*) > 0 for all *r*, *t* > 0, where *f*_*r*_ : [0, *∞*] → *∞*[0, *∞*] is defined by

(13) fr(t)=inf⁡x,y∈Br,||x−y||=t,α∈(0,1)((αg(x)+(1−α)g(y)−g(αx+(1−α)y))×(α(1−α))−1), ∀t≥0.

The function *f*_*r*_ is called the gauge of the uniform convexity of *g*. If *g* : *E* → ℝ is a convex function which is uniformly convex on bounded subsets, then

(14) g(αx+(1−α)y)≤αg(x)+(1−α)g(y) −α(1−α)fr(||x−y||)

for all *x*, *y* ∈ *B*_*r*_ and *α* ∈ (0,1), where *f*_*r*_ is the gauge of the uniform convexity of *g*.

Let *T* : *C* → *C* be a mapping. In this paper, we use *F*(*T*) to denote the fixed point set of *T*. *T* is said to be closed; if for any sequence {*x*_*n*_} ⊂ *C* such that lim⁡_*n*→*∞*_ *x*_*n*_ = *x*_0_ and lim⁡_*n*→*∞*_ *Tx*_*n*_ = *y*_0_, then *Tx*_0_ = *y*_0_.

Let *g* : *E* → ℝ be a proper, lower semicontinuous and convex function. Recall that *T* : *C* → *C* is said to be Bregman quasi-nonexpansive, if and only if

(15) F(T)≠∅, Dg(p,Tx)≤Dg(p,x),  ∀x∈C, p∈F(T).

A point *p* in *C* is said to be an asymptotic fixed point of *T* if and only if *C* contains a sequence {*x*_*n*_}, which converges weakly to *p* such that lim⁡_*n*→*∞*_ ||*x*_*n*_ − *Tx*_*n*_|| = 0. The set of asymptotic fixed points of *T* will be denoted by $\tilde{F}(T)$. *T* is said to be Bregman relatively nonexpansive if and only if $\tilde{F}(T)=F(T)\neq \emptyset$ and *D*_*g*_(*p*, *Tx*) ≤ *D*_*g*_(*p*, *x*) for all *x* ∈ *C* and *p* ∈ *F*(*T*).

Let *A* be a multivalued operator from *E* to E* with domain Dom⁡(*A*) = {*z* ∈ *E* : *Az* ≠ *∅*} and range Ran(*A*) = ∪{*Az* : *z* ∈ Dom⁡(*A*)}. An operator *A* is said to be monotone if and only if 〈*x*_1_ − *x*_2_, *y*_1_ − *y*_2_〉≥0 for each *x*_*i*_ ∈ Dom⁡(*A*) and *y*_*i*_ ∈ *Ax*_*i*_, *i* = 1,2. A monotone operator *A* is said to be maximal if its graph Grap(*A*) = {(*x*, *y*) : *y* ∈ *Ax*} is not properly contained in the graph of any other monotone operator. We know that if *A* is a maximal monotone operator, then *A* ^−1^(0) is closed and convex.

Let *E* be a reflexive, strictly convex, and smooth Banach space, and let *A* be a maximal monotone operator from *E* to E*. From Rockafellar [33], we find that *s* > 0 and *x* ∈ *E*; there exists a unique *x*_*s*_ ∈ *D*(*A*) such that ∇*gx* ∈ ∇*gx*_*s*_ + *s* *Ax*_*s*_. If *J*_*s*_ *x* = *x*_*s*_, then we can define a single-valued mapping *J*_*s*_ : *E* → Dom⁡(*A*) by *J*_*s*_ = (∇*g* + *sA*)^−1^∇*g* and such a *J*_*s*_ is called the resolvent of *A*. We know that *J*_*s*_ is closed and *A* ^−1^(0) = *F*(*J*_*s*_) for all *s* > 0. From [34], we know that *J*_*s*_ : *E* → Dom⁡(*A*) is a Bregman quasi-nonexpansive.

In this paper, we study a common solution problem based on Bregman projections. Strong convergence of the monotone projection algorithm for monotone operators and bifunctions is obtained in a reflexive Banach space.

In order to introduce our main results, we also need the following lemmas.

**Lemma 1 (see [34])**

Let *E* be a Banach space and let *g* : *E* → ℝ be a Gâteaux differentiable function which is uniformly convex on bounded subsets of *E*. Let {*x*_*n*_} and {*y*_*n*_} be bounded sequences in *E*. Then,

(16) $$\begin{array}{c} \underset{n\to \infty }{\lim}\;D_{g}\left(x_{n},y_{n}\right)=0\Leftrightarrow \underset{n\to \infty }{\lim}\;||x_{n}-y_{n}||=0. \end{array}$$

**Lemma 2 (see [35])**

Let *E* be a reflexive Banach space and let *g* : *E* → ℝ be a convex, continuous, strongly coercive, and Gâteaux differentiable function which is bounded on bounded subsets and uniformly convex on bounded subsets of *E*. Let *C* be a nonempty, closed, and convex subset of *E*. Let *T* : *C* → *C* be a Bregman relatively nonexpansive mapping. Then, *F*(*T*) is closed and convex.

**Lemma 3 (see [36])**

Let *E* be a reflexive Banach space and let *g* : *E* → ℝ be a convex, continuous, and strongly coercive function which is bounded on bounded subsets and uniformly convex on bounded subsets of *E*. Let *C* be a nonempty, closed, and convex subset of *E* and let *f* be a bifunction from *C* × *C* to ℝ satisfying (A1)–(A4). Let *r* > 0 and *x* ∈ *E*. Then, there exists *z* ∈ *C* such that *f*(*z*, *y*) + 1/*r*〈*y* − *z*, ∇*gz* − ∇*gx*〉 ≥ 0, for all *y* ∈ *C*. Define a mapping *S*_*r*_ : *E* → *C* by *S*_*r*_ *x* = {*z* ∈ *C* : *f*(*z*, *y*)+(1/*r*)〈*y* − *z*, ∇*gz* − ∇*x*〉, for all *y* ∈ *C*}. Then, the following conclusions hold:

(1) *S*_*r*_ is single-valued;
(2) *S*_*r*_ is a Bregman firmly nonexpansive-type mapping; that is, for all *x*, *y* ∈ *E*,
  (17) $$\begin{array}{c} \langle S_{r}x-S_{r}y,\nabla gS_{r}x-\nabla gS_{r}y\rangle \leq \langle S_{r}x-S_{r}y,\nabla x-\nabla gy\rangle ; \end{array}$$
(3) *F*(*S*_*r*_) = *EP*(*f*);
(4) *S*_*r*_ is Bregman quasi-nonexpansive;
(5) (18) $$\begin{array}{c} D_{g}\left(q,S_{r}x\right)+D_{g}\left(S_{r}x,x\right)\leq \phi \left(q,x\right),\;\;\;\forall q\in F\left(S_{r}\right); \end{array}$$
(6) *EP*(*f*) is closed and convex.

## 3. Main Results

**Theorem 4**

Let *E* be a reflexive Banach space and let *g* : *E* → *R* be a strongly coercive Bregman function which is bounded on bounded subsets and uniformly convex and smooth on bounded subsets of *E*. Let *C* be a nonempty, closed, and convex subset of *E* and let Λ be an index set. Let {*s*_*i*_} be a positive real number sequence. Let *f*_*i*_ be a bifunction from *C* × *C* to ℝ satisfying (A1)–(A4) and let *A*_*i*_ : *E* → E* be a maximal monotone operator such that Dom(*A*_*i*_) ⊂ *C* for every *i* ∈ Λ. Assume that the common solution set  *CSS* : = ∩_*i*∈Λ_ *A*_*i*_^−1^(0)⋂∩_*i*∈Λ_ *EF*(*f*_*i*_) is nonempty. Let {*x*_*n*_} be a sequence generated in the following manner:

(19) x0∈E  chosen  arbitrarily,C(1,i)=C,  C1=∩i∈ΛC(1,i),x1=ProjC1gx0,y(n,i)=∇g∗(α(n,i)∇g(xn)+(1−α(n,i))∇gJsiAixn),u(n,i)∈C    such  thatfi(u(n,i),y)+1r(n,i)〈y−u(n,i),∇g(u(n,i))−∇g(y(n,i))〉≥0,      ∀y∈C,C(n+1,i)={z∈C(n,i):Dg(z,u(n,i))≤Dg(z,xn)},Cn+1=∩i∈ΛC(n+1,i),xn+1=ProjCn+1gx1,

where ∇*g* is the right-hand derivative of *g*, *J*_*s*_*i*__^*A*_*i*_^ = (∇*g* + *s*_*i*_ *A*_*i*_)^−1^∇*g*, {*α*_(*n*,*i*)_} is a real sequence in [0,1] such that liminf⁡_*n*→*∞*_ *α*_(*n*,*i*)_(1 − *α*_(*n*,*i*)_) > 0, and {*r*_(*n*,*i*)_} is a real sequence in [*a*, *∞*), where *a* is some positive real number, for every *i* ∈ Λ. Then, the sequence {*x*_*n*_} converges strongly to *Proj*_*CSS*_^*g*^ *x*_1_, where *Proj*_*CSS*_^*g*^ is the Bregman projection from *E* onto *CSS*.

**Proof**

In view of Lemma 3, we find that the common solution set CSS is closed and convex. Next, we prove that *C*_*n*_ is closed and convex. It suffices to show that, for each fixed but arbitrary *i* ∈ Λ, *C*_(*n*,*i*)_ is closed and convex. This can be proved by induction into *n*. It is obvious that *C*_(1,*i*)_ = *C* is closed and convex. Assume that *C*_(*k*,*i*)_ is closed and convex for some *k* ≥ 1. For *z*_1_, *z*_2_ ∈ *C*_(*k*+1,*i*)_, we see that *z*_1_, *z*_2_ ∈ *C*_(*k*,*i*)_. It follows that *z* = *tz*_1_ + (1 − *t*)*z*_2_ ∈ *C*_(*k*,*i*)_, where *t* ∈ (0,1). Notice that

(20) $$\begin{array}{c} D_{g}\left(z_{1},u_{\left(k,i\right)}\right)\leq D_{g}\left(z_{1},x_{k}\right),\\ D_{g}\left(z_{2},u_{\left(k,i\right)}\right)\leq D_{g}\left(z_{2},x_{k}\right). \end{array}$$

The above inequalities are equivalent to

(21) 〈z1,∇g(x)−∇g(u(k,i))〉≤g(u(k,i))−g(xk) −〈u(k,i),∇g(u(k,i))〉+〈x,∇g(x)〉,〈z2,∇g(x)−∇g(u(k,i))〉≤g(u(k,i))−g(xk) −〈u(k,i),∇g(u(k,i))〉+〈x,∇g(x)〉.

Multiplying *t* and (1 − *t*) on both sides of (21), respectively, yields that

(22) 〈z,∇g(x)−∇g(u(k,i))〉≤g(u(k,i))−g(xk) −〈u(k,i),∇g(u(k,i))〉+〈x,∇g(x)〉.

That is,

(23) $$\begin{array}{c} D_{g}\left(z,u_{\left(k,i\right)}\right)\leq D_{g}\left(z,x_{k}\right), \end{array}$$

where *z* ∈ *C*_(*k*,*i*)_. This implies that *C*_(*k*+1,*i*)_ is closed and convex. We conclude that *C*_(*n*,*i*)_ is closed and convex. This in turn implies that *C*_*n*_ = ∩_*i*∈Λ_ *C*_(*n*,*i*)_ is closed and convex. This implies that Proj_*C*_*n*+1__^*g*^ *x*_1_ is well defined.

Next, we show that CSS ⊂ *C*_*n*_. CSS ⊂ *C*_1_ = *C* is clear. Suppose that CSS ⊂ *C*_(*k*,*i*)_ for some positive integer *k*. For any *w* ∈ CSS ⊂ *C*_(*k*,*i*)_, we see that

(24) Dg(w,u(k,i))=Dg(w,Sr(k,i)y(k,i))≤Dg(w,y(k,i))=Dg(w,∇g∗(α(k,i)∇g(xk)+(1−α(k,i))∇gJsiAixk))=V(w,α(k,i)∇g(xk)+(1−α(k,i))∇g(JsiAixk))=g(w)−α(k,i)〈w,∇g(xk)〉−(1−α(k,i)) ×〈w,∇g(JsiAixk)〉 +g∗(α(k,i)∇g(xk)+(1−α(k,i))∇g(JsiAixk))≤α(k,i)V(w,∇g(xk)) +(1−α(k,i))V(w,∇g(JsiAixk))≤α(k,i)Dg(w,xk)+(1−α(k,i))Dg(w,JsiAixk)≤Dg(w,xk),

which shows that *w* ∈ *C*_(*k*+1,*i*)_. This implies that CSS ⊂ *C*_(*n*,*i*)_. This yields that CSS ⊂ ∩_*i*∈Λ_ *C*_(*n*,*i*)_. This completes the proof that CSS ⊂ *C*_*n*_.

Next, we show that the sequence {*x*_*n*_} is bounded. It follows from (12) that

(25) Dg(xn,x1)≤Dg(ProjCSSgx1,x1)−Dg(ProjCSSgx1,xn)≤Dg(ProjCSSgx1,x1).

This implies that the sequence {*D*_*g*_(*x*_*n*_, *x*_1_)} is bounded. It follows that the sequence {*x*_*n*_} is also bounded. In view of the construction of the set *C*_*n*_, we find that *C*_*m*_ ⊂ *C*_*n*_ and *x*_*m*_ = proj_*C*_*m*__^*g*^ *x*_1_ ∈ *C*_*m*_ ⊂ *C*_*n*_ for any positive integer *m* ≥ *n*. It follows from (12) that

(26) Dg(xm,xn)=Dg(xm,ProjCngx1)≤Dg(xm,x1)−Dg(ProjCngx1,x1)=Dg(xm,x1)−Dg(xn,x1).

It follows that

(27) $$\begin{array}{c} D_{g}\left(x_{n},x_{1}\right)\leq D_{g}\left(x_{n},x_{1}\right)+D_{g}\left(x_{m},x_{n}\right)\leq D_{g}\left(x_{m},x_{1}\right). \end{array}$$

This shows that the sequence {*D*_*g*_(*x*_*n*_, *x*_1_)} is nondecreasing. Hence, the limit lim⁡_*n*→*∞*_ *D*_*g*_(*x*_*n*_, *x*_1_) exists. In view of *x*_*n*_ = Proj_*C*_*n*__^*g*^ *x*_1_, we find that

(28) $$\begin{array}{c} D_{g}\left(x_{n},x_{1}\right)\leq D_{g}\left(x_{n+1},x_{1}\right). \end{array}$$

This concludes that lim⁡_*n*→*∞*_ *D*_*g*_(*x*_*n*_, *x*_1_) exists. Let *m*, *n* → *∞* in (26); we find that *D*_*g*_(*x*_*m*_, *x*_*n*_) → 0. In view of Lemma 1, we obtain that ||*x*_*m*_ − *x*_*n*_|| → 0 as *m*, *n* → *∞*. This yields that {*x*_*n*_} is a Cauchy sequence. Since *C* is closed and convex, we see that there exists an $\bar{x}\in C$; that is,

(29) $$\begin{array}{c} \underset{n\to \infty }{\lim}||x_{n}-\bar{x}||=0. \end{array}$$

Next, we show that $\bar{x}\in \text{CSS}$. It follows from (26) that

(30) $$\begin{array}{c} \underset{n\to \infty }{\lim}\;D_{g}\left(x_{n+1},x_{n}\right)=0. \end{array}$$

Therefore, we obtain that

(31) $$\begin{array}{c} \underset{n\to \infty }{\lim}||x_{n+1}-x_{n}||=0. \end{array}$$

Since *x*_*n*+1_ ∈ *C*_*n*+1_, we find that

(32) $$\begin{array}{c} D_{g}\left(x_{n+1},u_{\left(n,i\right)}\right)\leq D_{g}\left(x_{n+1},x_{n}\right). \end{array}$$

In view of (30), we find that

(33) $$\begin{array}{c} \underset{n\to \infty }{\lim}\;D_{g}\left(x_{n+1},u_{\left(n,i\right)}\right)=0. \end{array}$$

It follows that

(34) $$\begin{array}{c} \underset{n\to \infty }{\lim}||x_{n+1}-u_{\left(n,i\right)}||=0. \end{array}$$

Notice that

(35) $$\begin{array}{c} ||x_{n}-u_{\left(n,i\right)}||\leq ||x_{n}-x_{n+1}||+||x_{n+1}-u_{\left(n,i\right)}||. \end{array}$$

It follows from (29) and (34) that lim⁡n→∞ ||xn-u(n,i)||=0. In view of Lemma 1, we find that

(36) $$\begin{array}{c} \underset{n\to \infty }{\lim}\;D_{g}\left(u_{\left(n,i\right)},x_{n}\right)=0. \end{array}$$

Since ∇*g* is uniformly norm-to-norm continuous on any bounded subset of *E*, we find that

(37) $$\begin{array}{c} \underset{n\to \infty }{\lim}||\nabla g\left(u_{\left(n,i\right)}\right)-\nabla g\left(x_{n}\right)||=0. \end{array}$$

Notice that

(38) |Dg(w,xn)−Dg(w,u(n,i))| =|Dg(u(n,i),xn)+w−u(n,i),〈∇g(u(n,i))−∇g(xn)〉| ≤|Dg(u(n,i),xn)|+||w−u(n,i)||||∇g(u(n,i))−∇g(xn)||.

It follows from (36) and (37) that

(39) $$\begin{array}{c} \underset{n\to \infty }{\lim}\left|D_{g}\left(w,x_{n}\right)-D_{g}\left(w,u_{\left(n,i\right)}\right)\right|=0. \end{array}$$

In view of (14), we find that

(40) Dg(w,u(n,i))=Dg(w,Sr(n,i)y(n,i))≤Dg(w,y(n,i))=Dg(w,∇g∗(α(n,i)∇g(xn)+(1−α(n,i))∇g(JsiAixn)))=V(w,α(n,i)∇g(xn)+(1−α(n,i))∇g(JsiAixn))=g(w)−α(n,i)〈w,∇g(xn)〉−(1−α(n,i)) ×〈w,∇g(JsiAixn)〉 +g∗(α(n,i)∇g(xn)+(1−α(n,i))∇g(JsiAixn))≤α(n,i)V(w,∇g(xn)) +(1−α(n,i))V(w,∇g(JsiAixn)) −ρ(||∇g(JsiAixn)−∇g(xn)||)≤α(n,i)Dg(w,xn)+(1−α(n,i))Dg(w,JsiAixn) −α(n,i)(1−α(n,i)) ×ρ(||∇g(JsiAixn)−∇g(xn)||)≤Dg(w,xn)−α(n,i)(1−α(n,i)) ×ρ(||∇g(JsiAixn)−∇g(xn)||),

where *ρ* is the gauge of the uniform convexity of g*. It follows that

(41) α(n,i)(1−α(n,i))ρ(||∇g(JsiAixn)−∇g(xn)||) ≤Dg(w,xn)−Dg(w,u(n,i)).

In view of the restrictions on the sequence {*α*_(*n*,*i*)_}, we find from (39) that lim⁡_*n*→*∞*_ ||∇*g*(*x*_*n*_)−∇*g*(*J*_*s*_*i*__^*A*_*i*_^ *x*_*n*_)|| = 0. Since ∇g* is uniformly norm-to-norm continuous on bounded subsets of E*, we obtain that

(42) $$\begin{array}{c} \underset{n\to \infty }{\lim}||x_{n}-J_{s_{i}}^{A_{i}}x_{n}||=0. \end{array}$$

Since *J*_*s*_*i*__^*A*_*i*_^ is closed Bregman quasi-nonexpansive, we find that $J_{s_{i}}^{A_{i}}\bar{x}=\bar{x}$. This proves that $\bar{x}\in \cap_{i\in \Lambda }A_{i}^{-1}(0)$.

Next, we show that $\bar{x}\in \cap_{i\in \Lambda }\text{EF}(f_{i})$. Notice that

(43) Dg(u(n,i),y(n,i))≤Dg(w,y(n,i))−Dg(w,u(n,i))≤Dg(w,xn)−Dg(w,u(n,i)).

It follows from (39) that

(44) $$\begin{array}{c} \underset{n\to \infty }{\lim}D_{g}\left(u_{\left(n,i\right)},y_{\left(n,i\right)}\right)=0. \end{array}$$

Therefore, we find that lim⁡_*n*→*∞*_||*u*_(*n*,*i*)_ − *y*_(*n*,*i*)_|| = 0. Since ∇*g* is uniformly norm-to-norm continuous on any bounded sets, we have lim⁡_*n*→*∞*_||∇*g*(*u*_(*n*,*i*)_)−∇(*y*_(*n*,*i*)_)|| = 0. From the assumption *r*_(*n*,*i*)_ ≥ *a*, we see that

(45) $$\begin{array}{c} \underset{n\to \infty }{\lim}\frac{||\nabla g\left(u_{\left(n,i\right)}\right)-\nabla g\left(y_{\left(n,i\right)}\right)||}{r_{\left(n,i\right)}}=0. \end{array}$$

In view of *u*_(*n*,*i*)_ = *S*_*r*_(*n*,*i*)__ *y*_(*n*,*i*)_, we see that

(46) fi(u(n,i),y)+1r(n,i)〈y−u(n,i),∇g(u(n,i))−∇g(y(n,i))〉≥0,∀y∈C.

It follows from (A2) that

(47) ||y−u(n,i)||||∇g(u(n,i))−∇g(y(n,i))||rn,i ≥1r(n,i)〈y−u(n,i),∇g(u(n,i))−∇g(y(n,i))〉 ≥fi(y,u(n,i)),      ∀y∈C.

In view of (A4), we find from (45) that

(48) $$\begin{array}{c} f_{i}\left(y,\bar{x}\right)\leq 0,\;\;\;\forall y\in C. \end{array}$$

For 0 < *t*_*i*_ < 1 and *y* ∈ *C*, define $y_{\left(t,i\right)}=t_{i}y+(1-t_{i})\bar{x}$. It follows that *y*_(*t*,*i*)_ ∈ *C*, which yields that $f(y_{\left(t,i\right)},\bar{x})\leq 0$. It follows from (A1) and (A4) that

(49) 0=f(y(t,i),y(t,i))≤tif(y(t,i),y)+(1−ti)f(y(t,i),x¯)≤tif(y(t,i),y).

That is,

(50) $$\begin{array}{c} f\left(y_{\left(t,i\right)},y\right)\geq 0. \end{array}$$

Letting *t*_*i*_ ↓ 0, we obtain from (A3) that $f_{i}(\bar{x},y)\geq 0$, for all *y* ∈ *C*. This implies that $\bar{x}\in \mathrm{EP}(f_{i})$ for every *i* ∈ Λ. This shows that $\bar{x}\in \text{CSS}$.

Finally, we prove that $\bar{x}={\text{Proj}}_{\text{CSS}}^{g}x_{1}$. In view of *x*_*n*_ = Proj_*C*_*n*__^*g*^ *x*_1_, we conclude that

(51) $$\begin{array}{c} \langle z-x_{n},\nabla g\left(x_{n}\right)-\nabla g\left(x_{1}\right)\rangle ,\;\forall z\in C_{n}. \end{array}$$

Since CSC ⊂ *C*_*n*_, we arrive at

(52) $$\begin{array}{c} \langle w-x_{n},\nabla g\left(x_{n}\right)-\nabla g\left(x_{1}\right)\rangle ,\;\forall w\in \text{CSC}. \end{array}$$

Letting *n* → *∞* in the above inequality, we see that

(53) $$\begin{array}{c} \langle w-\bar{x},\nabla g\left(\bar{x}\right)-\nabla g\left(x_{1}\right)\rangle \geq 0,\;\;\;\forall w\in \text{CSS}. \end{array}$$

This yields that $\bar{x}={\text{Proj}}_{\text{CSS}}^{g}x_{1}$. This completes the proof.

If the index set is singleton, then we have the following result.

**Corollary 5**

Let *E* be a reflexive Banach space and let *g* : *E* → *R* be a strongly coercive Bregman function which is bounded on bounded subsets and uniformly convex and smooth on bounded subsets of *E*. Let *C* be a nonempty, closed, and convex subset of *E* and let *s* be a positive real number. Let *f* be a bifunction from *C* × *C* to ℝ satisfying (A1)–(A4) and let *A* : *E* → E* be a maximal monotone operator such that Dom(*A*) ⊂ *C*. Assume that the common solution set *CSS* : = *A* ^−1^(0)⋂*EF*(*f*) is nonempty. Let {*x*_*n*_} be a sequence generated in the following manner:

(54) x0∈E  chosen  arbitrarily,C1=C,x1=ProjC1gx0,yn=∇g∗(αn∇g(xn)+(1−αn)∇g(JsAxn)),un∈C  such  that   f(un,y)+1rn〈y−un,∇g(un)−∇g(yn)〉≥0,∀y∈C,Cn+1={z∈Cn:Dg(z,un)≤Dg(z,xn)},xn+1=ProjCn+1gx1,

where ∇*g* is the right-hand derivative of *g*. *J*_*s*_^*A*^ = (∇*g*+*sA*)^−1^∇*g*, {*α*_*n*_} is a real sequence in [0,1] such that liminf⁡_*n*→*∞*_ *α*_*n*_(1 − *α*_*n*_) > 0, and {*r*_*n*_} is a real sequence in [*a*, *∞*), where *a* is some positive real number. Then, the sequence {*x*_*n*_} converges strongly to *Proj*_*CSS*_^*g*^ *x*_1_, where *Proj*_*CSS*_^*g*^ is the Bregman projection from *E* onto *CSS*.

**Remark 6**

Corollary 5 improves Theorem 4 of Qin et al. [24] in the following aspects: (1) the framework of spaces is relaxed; (2) a more general notion of Bregman function is considered instead of the generalized duality mapping; (3) a more general notion of Bregman projection is considered instead of the generalized projection operator; (4) families of operators extend from a finite family to an infinite family.

Finally, we give some results in the framework of Hilbert spaces.

**Theorem 7**

Let *E* be a Hilbert space and let *C* be a nonempty, closed, and convex subset of *E*. Let Λ be an index set. Let {*s*_*i*_} be a positive real number sequence. Let *f*_*i*_ be a bifunction from *C* × *C* to ℝ satisfying (A1)–(A4) and let *A*_*i*_ : *E* → *E* be a maximal monotone operator such that Dom(*A*_*i*_) ⊂ *C* for every *i* ∈ Λ. Assume that the common solution set *CSS* : = ∩_*i*∈Λ_ *A*_*i*_^−1^(0)⋂∩_*i*∈Λ_ *EF*(*f*_*i*_) is nonempty. Let {*x*_*n*_} be a sequence generated in the following manner:

(55) x0∈E  chosen  arbitrarily,C(1,i)=C,C1=∩i∈ΛC(1,i),x1=ProjC1x0,y(n,i)=α(n,i)xn+(1−α(n,i))JsiAixn,u(n,i)∈C  such  that   fi(u(n,i),y)+1r(n,i)〈y−u(n,i),u(n,i)−y(n,i)〉≥0,  ∀y∈C,C(n+1,i)={z∈C(n,i):||z−u(n,i)||2≤||z−xn||2},Cn+1=∩i∈ΛC(n+1,i),xn+1=ProjCn+1x1,

where *J*_*s*_*i*__^*A*_*i*_^ = (*I*+*s*_*i*_ *A*_*i*_)^−1^, {*α*_(*n*,*i*)_} is a real sequence in [0,1] such that liminf⁡_*n*→*∞*_ *α*_(*n*,*i*)_(1 − *α*_(*n*,*i*)_) > 0, and {*r*_(*n*,*i*)_} is a real sequence in [*a*, *∞*), where *a* is some positive real number, for every *i* ∈ Λ. Then, the sequence {*x*_*n*_} converges strongly to *Proj*_*CSS*_ *x*_1_, where *Proj*_*CSS*_ is the metric projection from *E* onto *CSS*.

**Proof**

Putting *g*(*x*) = (1/2)||*x*||^2^, we find that ∇*g*(*x*) = *x* for all *x* ∈ *E*. It follows that *D*_*g*_(*x*, *y*) = ||*x*−*y*||^2^ for all *x*, *y* ∈ *E*. From Theorem 4, we can immediately obtain the desired conclusion.

If the index set is singleton, then we have the following result.

**Corollary 8**

Let *E* be a Hilbert space and let *C* be a nonempty, closed, and convex subset of *E*. Let *s* be a positive real number. Let *f* be a bifunction from *C* × *C* to ℝ satisfying (A1)–(A4) and let *A* : *E* → *E* be a maximal monotone operator such that Dom(*A*) ⊂ *C*. Assume that the common solution set *CSS* : = *A* ^−1^(0)⋂*EF*(*f*) is nonempty. Let {*x*_*n*_} be a sequence generated in the following manner:

(56) x0∈E  chosen  arbitrarily,C1=C,x1=ProjC1x0,yn=αnxn+(1−αn)JsAxn,un∈C    such  that     f(un,y)+1rn〈y−un,un−yn〉≥0,      ∀y∈C,Cn+1={z∈Cn:||z−un||2≤||z−xn||2},xn+1=ProjCn+1x1,

where *J*_*s*_^*A*^ = (*I*+*sA*)^−1^, {*α*_*n*_} is a real sequence in [0,1] such that liminf⁡_*n*→*∞*_ *α*_*n*_(1 − *α*_*n*_) > 0 and {*r*_*n*_} is a real sequence in [*a*, *∞*), where *a* is some positive real number. Then, the sequence {*x*_*n*_} converges strongly to *Proj*_*CSS*_ *x*_1_, where *Proj*_*CSS*_ is the metric projection from *E* onto *CSS*.
